# Early Steps of the Biosynthesis of the Anticancer Antibiotic Pleurotin

**DOI:** 10.1021/acschembio.4c00599

**Published:** 2024-10-28

**Authors:** Jack A. Weaver, Duha Alkhder, Panward Prasongpholchai, Michaël D. Tadesse, Emmanuel L. de los Santos, Lijiang Song, Christophe Corre, Fabrizio Alberti

**Affiliations:** † School of Life Sciences, University of Warwick, Coventry, CV4 7AL, U.K.; ‡ Leicester Medical School, University of Leicester, Leicester, LE1 7RH, U.K.; § UCB Biopharma, 216 Bath Road, Slough, SL1 3WE, U.K.; ∥ Department of Chemistry, University of Warwick, Coventry, CV4 7AL, U.K.; ⊥ School of Life Sciences & Department of Chemistry, University of Warwick, Coventry, CV4 7AL, U.K.

## Abstract

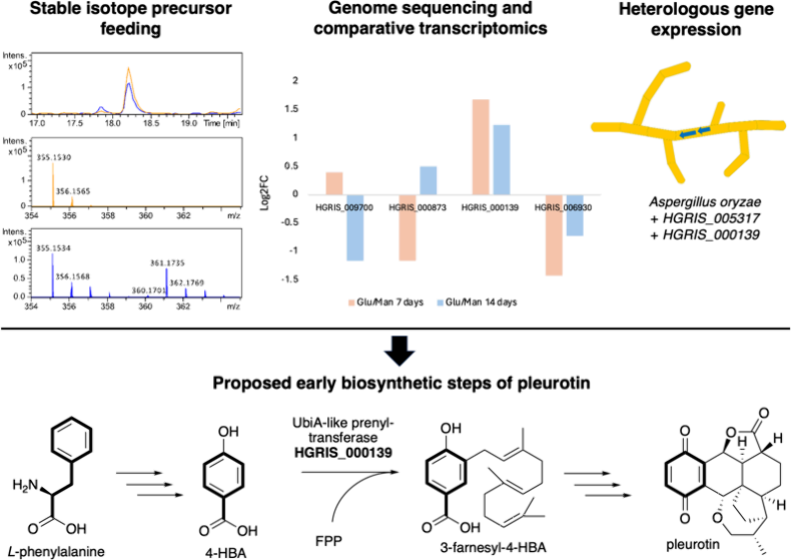

Pleurotin is a meroterpenoid specialized metabolite made by the fungus *Hohenbuehelia grisea*, and it is a lead anticancer molecule due to its irreversible inhibition of the thioredoxin-thioredoxin reductase system. Total synthesis of pleurotin has been achieved, including through a stereoselective route; however, its biosynthesis has not been characterized. In this study, we used isotope-labeled precursor feeding to show that the nonterpenoid quinone ring of pleurotin and its congeners is derived from phenylalanine. We sequenced the genome of *H. grisea* and used comparative transcriptomics to identify putative genes involved in pleurotin biosynthesis. We heterologously expressed a UbiA-like prenyltransferase from *H. grisea* that led to the accumulation of the first predicted pleurotin biosynthetic intermediate, 3-farnesyl-4-hydroxybenzoic acid. This work sets the foundation to fully elucidate the biosynthesis of pleurotin and its congeners, with long-term potential to optimize their production for therapeutic use and engineer the pathway toward the biosynthesis of valuable analogues.

## Introduction

Fungi are prolific producers of specialized metabolites, which are used for multiple applications including in medicine, such as β-lactam antimicrobials and cholesterol-lowering statins, and in enhancing crop yields, such as insecticide compounds made by entomopathogenic fungi.^1^ Among fungi, the mushroom-forming Basidiomycota are known to make a variety of structurally diverse compounds, including terpenoids, polyketides, and amino acid-derived specialized metabolites,^2^ some of which have high potential to be developed into drugs. However, this process is often hampered because of inherent difficulties connected with slow growth and challenging genetic engineering of Basidiomycota fungi.^3^ Understanding the biosynthesis of bioactive natural products from fungi of this division can allow us to direct it to the production of valuable analogues and congeners, as seen in the case of the pleuromutilin antibiotics.^4,5^

Among Basidiomycota fungi, the pleurotoid mushroom *Hohenbuehelia grisea* makes the anticancer antibiotic pleurotin, a meroterpenoid natural product that was first discovered in 1947 and shown to inhibit the growth of *Staphylococcus aureus*.^6^ Pleurotin was later proven to inhibit the growth of some fungi^7,8^ and to have antitumor activity through potent irreversible inhibition of the thioredoxin-thioredoxin reductase system, which makes it a lead anticancer compound.^9^

**Figure 1:**
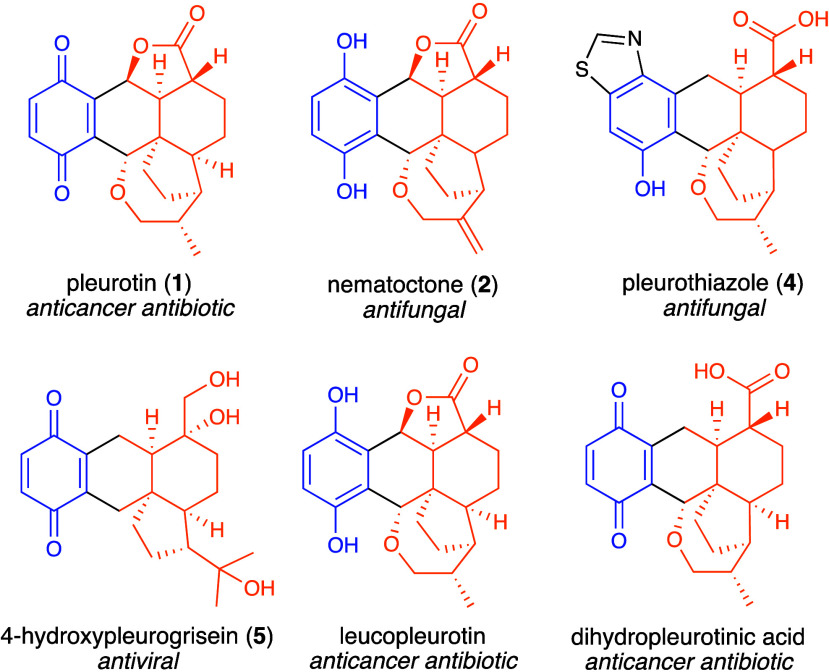
Structures of pleurotin and selected bioactive analogues. The terpenoid moiety is highlighted in orange, the nonterpenoid moiety in blue, the connecting bonds and additional groups in black.

Considerable work has emerged in the past years on the study of bioactive pleurotin congeners made by *H. grisea* (Figure 1) including the study of the antiviral 4-hydroxypleurogrisein^10^ and the isolation of cysteine-derived analogues of pleurotin.^11^ An optimized fermentation process for the production of pleurotin in high titers (more than 300 mg l^–1^) has been developed.^12^ The total synthesis of (±)-pleurotin has been achieved, through 26^13^ and 13 linear steps,^14^ as well as of its congeners (±)-pleurogrisein and (±)-4-hydroxypleurogrisein.^15^ Importantly, the stereoselective syntheses of (−)-pleurotin and its congeners (+)-leucopleurotin, (+)-dihydropleurotinic acid, and (+)-leucopleurotinic acid were recently reported in 15–16 steps,^16^ paving the way for the synthesis of new stereoselective pleurotin analogues.

Preliminary studies on the biosynthesis of pleurotin were conducted by Arigoni’s group through feeding of the fungus with isotope-labeled predicted precursors and intermediates, suggesting that pleurotin may derive from farnesylhydroquinone through several steps of cyclization, rearrangement, and oxidations.^17−19^ The exact sequence of reactions that lead to pleurotin is still unknown, and so are the enzymes involved in the biosynthetic pathway.

In this study, we set out to investigate the origin of the quinone ring of pleurotin by means of isotope-labeled precursor feeding. We also sequenced the genome of the pleurotin-producing fungus *H. grisea* and performed comparative transcriptomics analysis to pinpoint candidate biosynthetic genes. Characterization of a UbiA-like prenyltransferase (PTase) led us to isolate the first predicted intermediate in pleurotin biosynthesis. This work sets the foundation to characterize the biosynthetic pathway to pleurotin and its valuable congeners.

## Results and Discussion

### Isolation and Structural Characterization of Pleurotin

First, we cultured *Hohenbuehelia grisea* (strain ATCC 60515) in YM glucose to confirm production of pleurotin (**1**) from this strain. Metabolite extracts were analyzed through liquid chromatography-high-resolution mass spectrometry (LC-HRMS), in which extracted ion chromatogram at *m*/*z* 355.1545 (calculated for [M + H]^+^, where M = C_21_H_22_O_5_) showed a main species at retention time 20.5′ and a minor one at retention time 18.2′ (Figure S1). Based on the literature, we hypothesized that the minor product is likely to be pleurotin’s structural isomer nematoctone (**2**), which is reported to be produced in sub-milligram per liter level by pleurotin-producing fungi.^10^ In order to unequivocally confirm the identity of pleurotin, we scaled up cultures of *H. grisea* in YM glucose and used a combination of flash chromatography and HPLC to purify **1**. Structural characterization was achieved through ^1^H NMR spectroscopy (Figure S2), which was in agreement with literature data reported for **1**.^16^

### Biosynthetic Origin of the Quinone Ring of Pleurotin and Its Congeners

Meroterpenoids are hybrid natural products that include a terpenoid moiety and a nonterpenoid portion (see Figure 1). The nonterpenoid side of fungal meroterpenoids often includes a polyketide moiety, e.g., 5-methylorsellinic acid in mycophenolic acid,^20^ which is made by an iterative type-I polyketide synthase (PKS). However, fungal meroterpenoid biosynthetic pathways can sometimes use unusual starter units, such as nicotinyl-CoA in pyripyropene A biosynthesis.^21^ They can also be made independently of PKSs and use 4-hydroxybenzoic acid (4-HBA) as a precursor for their nonterpenoid moiety. For instance, the biosynthesis of antroquinonol^22^ and vibralactone^23^ involves the use of 4-HBA that can either be made through the endogenous shikimate pathway via chorismate or through exogenous phenylalanine.

**Figure 2:**
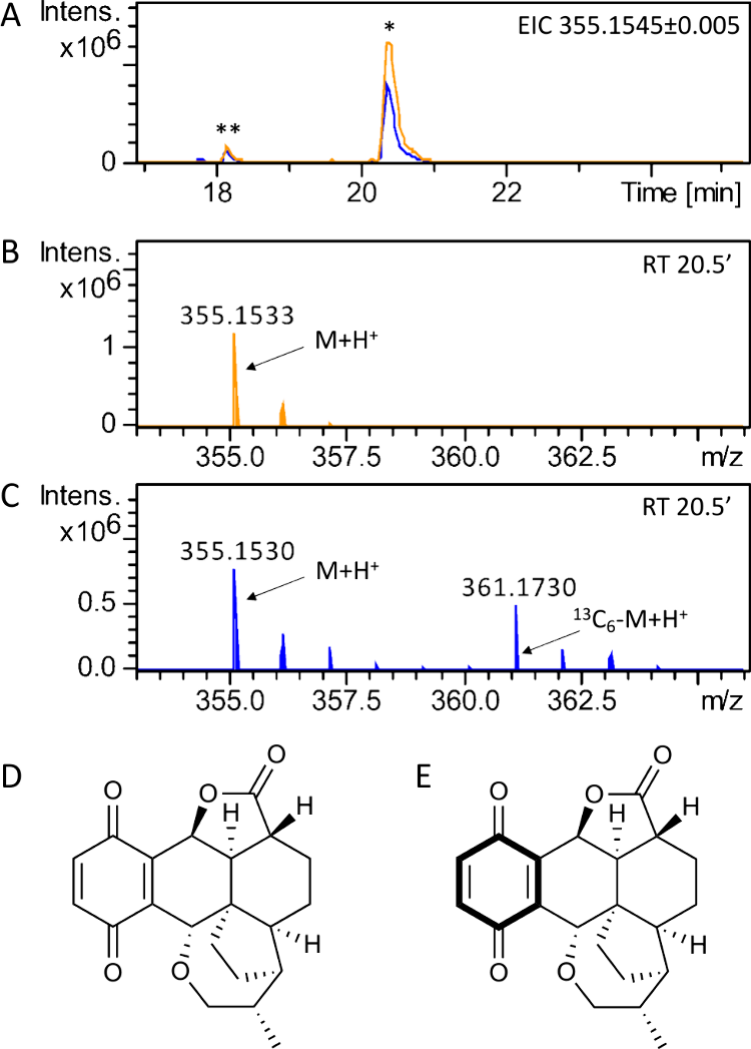
LC-HRMS detection of pleurotin (**1**) from *H. grisea* fungal extract. (**A**) Extracted ion chromatogram in positive mode at *m*/*z* = 355.1545 ± 0.005 is shown, highlighting accumulation of pleurotin in *H. grisea* crude extracts grown in standard *L*-phenylalanine (trace in orange) and in *L*-phenyl-^13^C_9_-alanine (trace in blue). One major peak (*) at retention time 20.5′ is seen, corresponding to pleurotin (**1**), based on NMR characterization of the purified compound; one additional peak (**) at retention time 18.2′ is seen, likely corresponding to nematoctone (**2**).^10^ (**B**) Mass spectrum of pleurotin (M = C_21_H_22_O_5_) detected at retention time 20.5′; *m*/*z* calculated for [M + H]^+^ = 355.1545. (**C**) Mass spectrum of pleurotin and ^13^C_6_-pleurotin detected at retention time 20.5′; *m*/*z* calculated for [^13^C_6_-M + H]^+^ = 361.1746. (**D**) Pleurotin. (**E**) ^13^C_6_-pleurotin.

Arigoni’s group performed preliminary studies on pleurotin biosynthesis through incorporation of [1-^13^C]- and [1,2-^13^C_2_]-acetate to show that **1** includes a C_15_ terpenoid side that derives from the mevalonate pathway, whereas its quinone ring was shown to derive from 4-hydroxybenzoic acid (4-HBA) through incorporation of a deuterated analogue of it.^18,19^ In order to shed light on the biosynthetic origin of the nonterpenoid side of pleurotin, we decided to test whether phenylalanine (**3**) can provide the quinone ring of **1**. We fed cultures of *H. grisea* with *L*-phenyl-^13^C_9_-alanine and analyzed ethyl acetate crude metabolite extracts through LC-HRMS, using *H. grisea* grown in standard *L*-phenylalanine as a control (Figure 2). The extract of *H. grisea* fed with *L*-phenyl-^13^C_9_-alanine showed a species with *m*/*z* 361.1730 (calculated *m*/*z* of 361.1746) at retention time 20.5′ (Figure 2C) corresponding to a six-Da increase compared to the pleurotin peak with *m*/*z* 355.1530 (calculated *m*/*z* of 355.1545) and absent in *H. grisea* grown in standard *L*-phenylalanine (Figure 2B). MS^2^ spectra were analyzed to further confirm the incorporation of the six heavy carbons in **1** (Figure S3). We also investigated the incorporation of the heavy carbons provided by *L*-phenyl-^13^C_9_-alanine in other pleurotin-related metabolites that we could observe within the extract. We were able to detect the same six-Da shift for the additional species with *m*/*z* 355.1530 at retention time 18.2′ (Figure S4), a species with *m*/*z* 400.1568 at retention time 19.4′ (Figure S5), and one with *m*/*z* 361.1996 at retention time 16.3′ (Figure S6), which returned predicted molecular formulas corresponding to those of the known pleurotin congeners nematoctone (**2**),^10^ pleurothiazole (**4**),^11^ and 4-hydroxypleurogrisein (**5**),^10^ respectively. High percentages of ^13^C incorporation from *L*-phenyl-^13^C_9_-alanine into **1**, **2**, **4**, and **5** were detected, ranging from 63 to 68% compared to the corresponding ^12^C-containing species (Table S1).

**Scheme 1:**
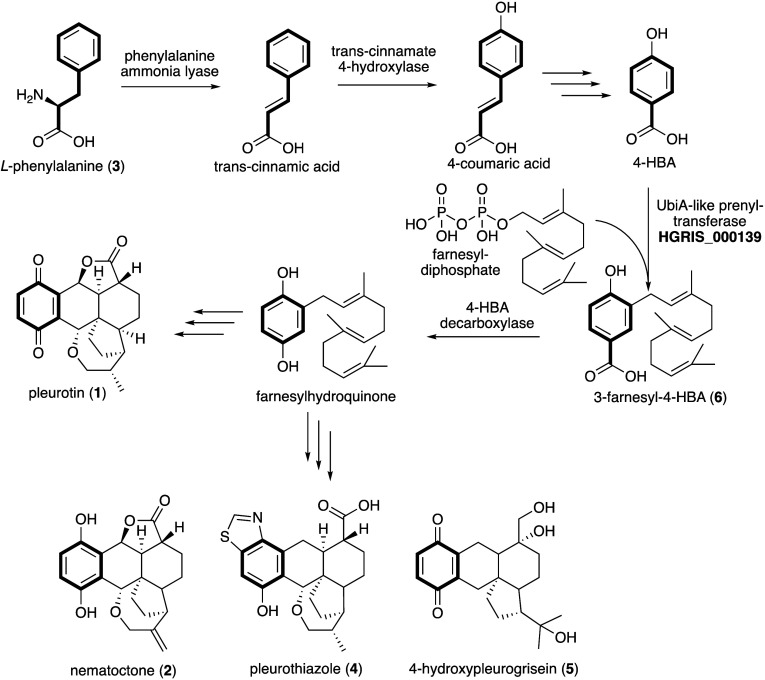
Proposed Biosynthesis of Pleurotin and Selected Congeners in *H. grisea*

Based on stable isotope feeding, we propose that *L*-phenylalanine (**3**) can provide the quinone ring of **1** and of its congeners **2**, **4**, and **5** (Scheme 1). A phenylalanine ammonia lyase, which is sometimes found in fungal meroterpenoid BGCs,^24,25^ is likely to convert **3** into *trans*-cinnamic acid, which can then undergo hydroxylation to make 4-coumaric acid, followed by side-chain degradation to give 4-HBA. We then predict the farnesylation of 4-HBA to be catalyzed by a UbiA-like PTase, a widely characterized enzyme class involved in the biosynthesis of fungal meroterpenoids such as mycophenolic acid,^20^ anditomin,^26^ ascochlorin, and ascofuranone,^27^ to name a few.

### Whole-Genome Sequencing of *Hohenbuehelia grisea*

Since no genome sequence was publicly available for pleurotin-producing fungi, we next aimed to sequence the genome of *H. grisea* ATCC 60515, so that it would serve as a framework to look for the biosynthetic genes involved in the production of pleurotin. Genomic DNA from *H. grisea* was purified and subjected to whole-genome sequencing using a combination of nanopore long-read sequencing and Illumina short-read sequencing, as well as Illumina-sequenced mRNA for improved gene annotation for Funannotate training.^28^ The assembled genome was deposited on NCBI under accession number JASNQZ000000000, BioProject: PRJNA956249. The genome had a total length of 38.87 Mb and was composed of 25 contigs (N_50_ 2.88 Mb). Full assembly statistics are reported in Table S2. The completeness of the genome assembly and annotation were assessed using BUSCO, which returned a high score of 97.3% for the genomic scaffold and of 94.3% for the predicted proteome (see full BUSCO results in Table S3). A total of 14,934 genes were predicted to be in the genome, out of which 14,602 were predicted to be protein-coding. On average, each gene was anticipated to include 6.72 exons. Additionally, 332 tRNA genes were predicted.

### Analysis of *Hohenbuehelia grisea* Secondary Metabolite Biosynthetic Gene Clusters

The biosynthetic enzymes that produce fungal meroterpenoids are often encoded by genes that are colocalized in biosynthetic gene clusters (BGCs).^29^ We therefore analyzed the assembled and annotated genome of *H. grisea* for the presence of secondary metabolite gene clusters using fungiSMASH,^30^ which returned 21 predicted BGCs (see Table 1). The biosynthesis of meroterpenoid natural products like **1** generally involves signature biosynthetic genes such as the aforementioned UbiA-like PTase for prenylation of a nonterpenoid moiety, as well as a transmembrane terpene cyclase (TC) for cyclization. A PKS can also be present in the BGC if the meroterpenoid includes a polyketide nonterpenoid moiety.^29^

**Table 1:** Secondary metabolite analysis of the *H. grisea* genome performed through FungiSMASH v 7.0^a^

Contig	Region	Type	From	To	Most similar known cluster	Similarity	Terpene synthase
utg3	1.1	terpene	1,269,614	1,293,566			HGRIS_005924
	1.2	terpene	1,817,696	1,844,739			HGRIS_006127
	1.3	NRPS-like	2,010,069	2,073,467	HEx-pks15 polyketide	28%	
utg5	2.1	terpene	1,587,531	1,608,732	(+)-δ-cadinol	100%	HGRIS_011751
utg12	4.1	NRPS-like	801,553	845,390			
utg13	5.1	terpene	655,545	676,459			HGRIS_001238
	5.2	terpene	763,776	784,976	(+)-δ-cadinol	100%	HGRIS_000005
utg18	7.1	NI-siderophore	689,929	725,511			
	7.2	terpene	833,379	854,574			HGRIS_003606
utg27	8.1	terpene	2,635,852	2,657,827			HGRIS_005332
utg32	11.1	fungal-RiPP-like	1,415,611	1,481,471			
utg42	14.1	terpene	572,644	593,839			HGRIS_010725
	14.2	T1PKS	1,032,220	1,079,105			
	14.3	terpene	1,153,317	1,176,290			HGRIS_010919
utg75	15.1	fungal-RiPP-like, T1PKS	226,773	332,576			
	15.2	terpene	1,746,623	1,769,494	(+)-δ-cadinol	100%	HGRIS_012714
	15.3	NRPS	1,907,631	1,956,146			
	15.4	terpene	4,193,940	4,216,534			HGRIS_013719
utg98	17.1	terpene	339,523	361,491			HGRIS_014814
utg129	18.1	terpene	533,335	554,508	(+)-δ-cadinol	100%	HGRIS_000941
	18.2	T1PKS	566,247	612,378			

a NRPS = non-ribosomal peptide synthetase; NI-siderophore = NRPS-independent, IucA/IucC-like siderophore; RiPP = ribosomally synthesised and post-translationally modified peptide; T1PKS = type I polyketide synthase.

Upon close inspection of the BGCs detected through fungiSMASH in the genome of *H. grisea*, including the 12 terpene BGCs, no UbiA-like PTase gene could be identified. We therefore searched for UbiA-like PTases within the genome of *H. grisea* through local BlastP against the predicted proteome of the fungus, using the sequence of the *Saccharomyces cerevisiae* UbiA-like PTase COQ2 (para hydroxybenzoate:polyprenyltransferase)^31^ as the query. We found four high-scoring homologous predicted proteins to COQ2 in *H. grisea*, HGRIS_009700, HGRIS_000873, HGRIS_000139, and HGRIS_006930, which share homology with COQ2 of 45%, 33%, 33%, and 36%, respectively (see Table S4). Similarly, we searched for homologues of the FAD-binding mono-oxygenase VibMO1, which is responsible for the oxidative decarboxylation of prenyl 4-HBA as part of vibralactone biosynthesis in the Basidiomycota fungus *Boreostereum vibrans*.^32^ An enzyme homologous to VibMO1 can be predicted to decarboxylate 3-farnesyl-4-HBA into farnesylhydroquinone in the early steps of pleurotin biosynthesis (Scheme 1). Seven high-scoring homologous proteins to VibMO1 were found in *H. grisea*, HGRIS_002929, HGRIS_001008, HGRIS_001007, HGRIS_004802, HGRIS_014061, HGRIS_008360, and HGRIS_010717 (Table S5). None of these appeared to be located in the putative terpenoid BGCs detected through FungiSMASH.

### Comparative Transcriptomics Analysis Enables the Shortlisting of Putative Pleurotin Biosynthesis Genes

In order to investigate the involvement of the putative biosynthetic genes found through FungiSMASH and local BlastP in the biosynthesis of **1**, we performed a comparative transcriptomics analysis in *H. grisea* cultures that exhibited differential pleurotin production. Specifically, cultures of *H. grisea* were grown in a shake-flask in media containing five different carbon sources (glucose, mannitol, fructose, galactose, and lactose - based on Robbins et al.^6^) in parallel. Ethyl acetate metabolite extracts arising from 2-week-old fungal cultures were analyzed using LC-HRMS, showing that only cultures grown in glucose as the carbon source were able to produce **1** in appreciable amounts, which was readily detected as a peak with *m*/*z* 355.1532 at retention time 20.5′ (Figure S7). Fungi grown in fructose as the carbon source showed limited production of pleurotin, whereas cultures grown in mannitol, fructose, and galactose did not produce pleurotin at all.

Once differential production of **1** could be ascertained in different culturing media, glucose and mannitol were picked as the two carbon sources to perform RNaseq analysis at two time points, seven and 14 days, based on the observation that accumulation of **1** could be detected starting from as early as one-week when culturing *H. grisea* in YM glucose. On day seven, 512 genes were overexpressed in glucose-grown mycelia compared to mannitol-grown mycelia (3.43% of the total number of genes) (Supplementary Data set 1). At day 14, the number of overexpressed genes between glucose-grown and mannitol-grown mycelia had increased to 948 (6.35% of the total number of genes). The expression of the *H. grisea* UbiA-like PTase homologue genes was examined, which showed that only *HGRIS_000139* was upregulated during production of **1** at 7 days (showing a Log_2_FC of 1.68), whereas the three other PTases were downregulated in glucose-grown compared to mannitol-grown fungi at both time points (Figure S8). Looking at the *H. grisea* homologues of FAD-binding mono-oxygenase VibMO1, only gene *HGRIS_001007* appeared to be upregulated in glucose-grown compared to mannitol-grown fungi at day seven, with a Log_2_FC of 2.20 (Figure S9).

Interestingly, most terpene synthases identified to be within *H. grisea* BGCs from FungiSMASH were downregulated when pleurotin was produced (Figure S10), with the only exception of HGRIS_005332, which was upregulated at day 14 with a Log_2_FC of 1.54. However, BlastP analysis of the clustered genes at region 8.1 (Table S6) excluded any involvement of nearby genes in HGRIS_005332 in the biosynthesis of secondary metabolites.

### The UbiA-like PTase HGRIS_000139 Catalyzes Farnesylation of 4-HBA to Make 3-Farnesyl-4-HBA

**Figure 3:**
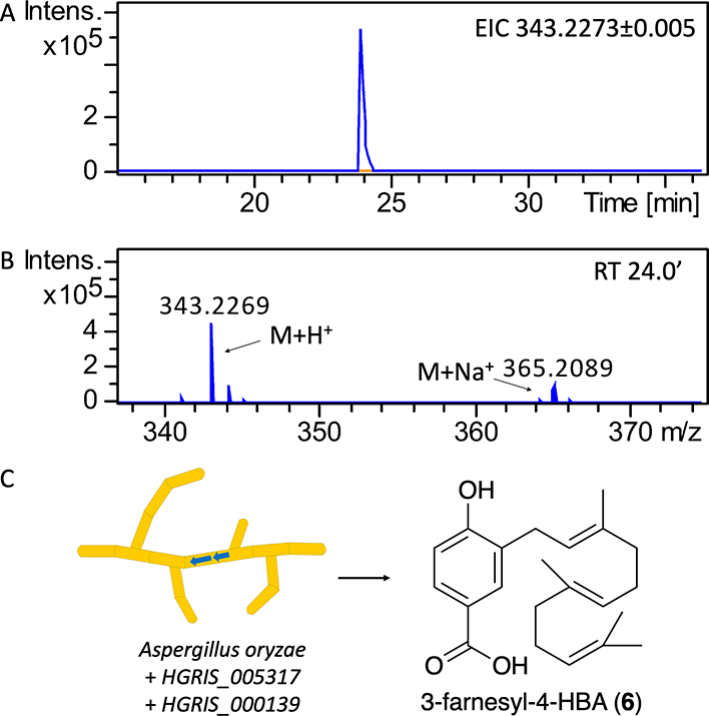
LC-HRMS detection of 3-farnesyl-4-HBA (**6**) in *A. oryzae* DA1. (**A**) Extracted ion chromatogram in positive mode at *m*/*z* = 343.2273 ± 0.005 is shown, highlighting accumulation of **6** in *A. oryzae* DA1 crude metabolite extracts (trace in blue) and its absence in *A. oryzae* NSAR1 (trace in orange). (**B**) Mass spectrum of **6** (M = C_22_H_30_O_3_) detected at retention time 24.0′. (**C**) Schematic representation of the heterologous expression of *HGRIS_005317* and *HGRIS_000139* in *A. oryzae* leading to the accumulation of **6**.

Since the putative UbiA-like PTase *HGRIS_000139* was upregulated during pleurotin production, we aimed to investigate its function by expressing it heterologously in *Aspergillus oryzae* NSAR1.^33^ We also decided to include a copy of the *H. grisea* farnesyl pyrophosphate synthase (FPPS) gene *HGRIS_005317*, to provide increased amounts of FPP for a suitable precursor supply. To this aim, we amplified the intron-less sequences of both *HGRIS_000139* and *HGRIS_005317* from the cDNA of *H. grisea* (Figure S11) and used them to assemble an expression vector based on the pTYGSarg plasmid backbone,^34^ named pDA001 (Figure S12). We transformed pDA001 into *A. oryzae* NSAR1 and confirmed heterologous gene insertion in *A. oryzae* DA1 through PCR amplification of *HGRIS_005317* and *HGRIS_000139* (Figures S13, S14). Metabolite analysis was performed through LC-HRMS to compare the crude extracts of *A. oryzae* DA1 and *A. oryzae* NSAR1, which highlighted the accumulation of a species with *m*/*z* 343.2269 in *A. oryzae* DA1, absent in the recipient strain *A. oryzae* NSAR1 (Figure 3). Prediction of the most likely molecular formula suggested a species with formula C_22_H_30_O_3_ (calculated for M[C_22_H_30_O_3_]^+^ = 343.2273), which was consistent with the formula of 3-farnesyl-4-hydroxybenzoic acid (3-farnesyl-4-HBA) (**6**). Purification of **6** was achieved using a combination of flash chromatography and HPLC, and its structure was confirmed through ^1^H NMR spectroscopy (Figure S15), which was in agreement with literature data reported for **6**.^24,35^ We therefore propose that HGRIS_000139 is a UbiA-like PTase that catalyzes the farnesylation of 4-HBA in *H. grisea* to give the first predicted pleurotin intermediate **6**.

## Conclusions

In summary, we characterized the early steps of pleurotin biosynthesis, showing that *L*-phenylalanine can provide the quinone ring of **1**, likely via 4-HBA, and that the farnesylation of 4-HBA catalyzed by the UbiA-like aPTase HGRIS_000139 leads to the first predicted pathway intermediate 3-farnesyl-4-HBA. We also sequenced the genome of the pleurotin-producing fungus *H. grisea*, providing a scaffold for the identification of the other pleurotin biosynthetic genes. FungiSMASH detection of putative BGCs did not conclusively point toward a candidate pleurotin BGC. Examples are known of fungal meroterpenoids that are produced by enzymes that are not colocalized, such as vibralactone,^36^ or are made by enzymes encoded by genes that are split across two BGCs, such as ascofuranone.^27^ Comparative transcriptomics analysis allowed us to generate a list of candidate biosynthetic genes that can be further characterized to determine their involvement in pleurotin biosynthesis and obtain a full picture of the pathway.

## Materials and Methods

The complete materials and methods section is included in the Supporting Information.

## Data Availability

The data supporting this article have been included as part of the Supporting Information and Supplementary Data set 1. The assembled genome was deposited on NCBI under accession number JASNQZ000000000, BioProject: PRJNA956249.

## Supporting Information Available

Differential gene expression in glucose- and mannitol-grown mycelia at two time points, 7 and 14 days (XLSX)
Materials and methods
Figure S1. LC-HRMS detection of pleurotin
Figure S2. ^1^H NMR spectrum of 1 in CDCl_3_ (400 MHz)
Figure S3. MS^2^ spectra of H. grisea crude extracts grown in standard l-phenylalanine and in l-phenyl-^13^C_9_-alanine
Figure S4. LC-HRMS detection of nematoctone (2)
Figure S5. LC-HRMS detection of pleurothiazole (4)
Figure S6. LC-HRMS detection of 4-hydroxypleurogrisein (5)
Table S1. Percentage of ^13^C incorporation from l-phenyl-^13^C_9_-alanine into pleurotin and three congeners
Table S2. Assembly statistics of the genome of H. grisea
Table S3. BUSCO analysis of H. grisea to assess the quality of genome scaffold and predicted proteome
Table S4. BlastP analysis of the H. grisea predicted proteome using *Saccharomyces cerevisiae* COQ2 as a query
Table S5. BlastP analysis of the H. grisea predicted proteome using Boreostereum vibrans VibMO1 as a query
Figure S7. LC-HRMS detection of pleurotin from H. grisea cultures grown in YM broth containing different carbon sources
Figure S8. Relative gene expression levels of H. grisea COQ2 homologues under pleurotin producing and nonproducing fermentation conditions
Figure S9. Relative gene expression levels of H. grisea VibMO1 homologues under pleurotin producing and nonproducing fermentation conditions
Figure S10. Relative gene expression levels of H. grisea terpene synthases from FungiSMASH BGCs under pleurotin producing and nonproducing fermentation conditions
Table S6. BlastP analysis of the translated genes from the BGC in region 8.1 from the genome of H. grisea
Figure S11. PCR amplification of genes HGRIS_005317 and HGRIS_000139
Figure S12. Plasmid map of pDA001
Figure S13. PCR amplification screening of gene HGRIS_005317
Figure S14. PCR amplification screening of gene HGRIS_000139
Figure S15. ^1^H NMR spectrum of 6 in CDCl_3_ (400 MHz)
Table S7. List of oligonucleotides and DNA sequences used in this work (PDF)

## Abbreviations

4-HBA: 4-hydroxybenzoic acid
BGC: biosynthetic gene cluster
LC-HRMS: liquid chromatography-high-resolution mass spectrometry
FPP: farnesyl pyrophosphate
FPPS: farnesyl pyrophosphate synthase
NI-siderophore: NRPS-independent, IucA/IucC-like siderophore
NMR: Nuclear magnetic resonance
NRPS: nonribosomal peptide synthetase
PKS: polyketide synthase
PTase: prenyltransferase
RiPP: ribosomally synthesized and post-translationally modified peptide
RNAseq: RNA sequencing
